# Design, Synthesis and Evaluation of Novel Antimicrobial Polymers Based on the Inclusion of Polyethylene Glycol/TiO_2_ Nanocomposites in Cyclodextrin as Drug Carriers for Sulfaguanidine

**DOI:** 10.3390/polym14020227

**Published:** 2022-01-06

**Authors:** Hemat M. Dardeer, Arafat Toghan, Magdi E. A. Zaki, Rokaia B. Elamary

**Affiliations:** 1 Chemistry Department, Faculty of Science, South Valley University, Qena 83523, Egypt; prof_hemat@sci.svu.edu.eg; 2 Chemistry Department, College of Science, Imam Mohammad Ibn Saud Islamic University (IMSIU), Riyadh 11623, Saudi Arabia; Mezaki@imamu.edu.sa; 3 Botany and Microbiology Department, Faculty of Science, South Valley University, Qena 83523, Egypt; rokia_bahgat@sci.svu.edu.eg

**Keywords:** cyclodextrin inclusion complexes, PEG/TiO_2_ nanocomposite polymers, drug delivery, sulfaguanidine, antibiotics

## Abstract

Polymers and their composites have recently attracted attention in both pharmaceutical and biomedical applications. Polyethylene glycol (PEG) is a versatile polymer extensively used in medicine. Herein, three novel PEG-based polymers that are pseudopolyrotaxane (PEG/α-CD) (**1**), titania–nanocomposite (PEG/TiO_2_NPs) (**2**), and pseudopolyrotaxane–titania–nanocomposite (PEG/α-CD/TiO_2_NPs) (**3**), were synthesized and characterized. The chemical structure, surface morphology, and optical properties of the newly materials were examined by FT-IR, ^1^H-NMR, SEM, and UV–Vis., respectively. The prepared polymers were used as drug carriers of sulfaguanidine as PEG/α-CD/Drug (**4**), PEG/TiO_2_NPs/Drug (**5**), and PEG/α-CD/TiO_2_NPs/Drug (**6**). The influence of these drug-carrying formulations on the physical and chemical characteristics of sulfaguanidine including pharmacokinetic response, solubility, and tissue penetration was explored. Evaluation of the antibacterial and antibiofilm effect of sulfaguanidine was tested before and after loading onto the prepared polymers against some Gram-negative and positive bacteria (*E. coli*, *Pseudomonas aeruginosa*, and *Staphylococcus aureus* (MRSA)), as well. The results of this work turned out to be very promising as they confirmed that loading sulfaguanidine to the newly designed polymers not only showed superior antibacterial and antibiofilm efficacy compared to the pure drug, but also modified the properties of the sulfaguanidine drug itself.

## 1. Introduction

The recent technological development can be considered a double-edged sword due to its negative impact on human health, especially the issue of environmental pollution and the spread of many chronic diseases such as autoimmune, metabolism, and cancer [1,2,3]. Unfortunately, some of these diseases are still difficult to treat by conventional pharmacotherapy that relies primarily on many factors such as solubility, molecular weight, and hydrogen bonds, all of which can impede the achievement of a therapeutic response. Although some cases can be treated using traditional methods, they are accompanied by a number of strong side effects [4]. However, scientists are always trying their best to find solutions to overcome the side effects of these treatments. Thus, there are various methods to reduce these side effects as much as possible, such as modifying the pharmacokinetic properties of drugs [5,6] and targeting the tumor tissue by releasing the drug at this site [7,8,9]. In addition, the radiotherapy method is available [10,11,12,13], but this approach remains a challenging approach, due to the change in the tumor type during the treatment. Hence, it is necessary to find newly safe methods to increase the effectiveness of some drugs by enhancement of their bioavailability and stability. and at the same time minimizing their side effects. For these methods, using some materials as drug carrier systems, cyclodextrins (CDs) have been of interest in the pharmaceutical industry [14,15,16,17]. CDs are used as complexing agents by forming inclusion complexes with different poorly soluble drugs, because of their availability, water-solubility, and biocompatibility [18]. Moreover, CDs can be used to reduce gastrointestinal drug irritation, convert liquid drugs into microcrystalline or amorphous powder, and prevent drug–drug and drug–excipient interactions [19]. CDs are cyclic oligosaccharides consisting of glucopyranose units, and the most common types are (α-CD), (β-CD), and (γ-CD) [20]. The mechanism of CDs depends on the formation of inclusion complexes with, or in, organic molecules due to their unique chemical structures which look similar to truncated cones, with external surfaces having hydrophilic properties, and the internal surface having hydrophobic properties [21,22]. Therefore, CDs can be used as targeting agents and in drug-carrier applications [23].

Polymers have played a vital role in the conventional formulations; they are used as film-coating agents in tablets, a binding agent in capsules, and as viscosity enhancers in emulsions. Many types of polymers are used in drug formulation, such as poly (N-vinyl pyrrolidone) and poly (ethylene glycol) PEG. Pharmacologists and scientists use polymers to control drug release [24]. The rate of the drug release from a matrix product is based on the initial drug concentration and relaxation of the polymer chains which overall shows a continued release characteristic [25]. Currently, two types of biodegradable polymers, natural polymers and synthetic polymers, have been used as drug-delivery systems [26]. Collagen and gelatin are two natural biodegradable polymers that are mostly used in drugs [26,27]. These advanced systems play a promising role in improving bioavailability and minimizing side effects and other types of inconveniences caused to the patients.

Polyrotaxane is one of the supramolecular polymers in that several of the macrocyclic molecules are threaded onto a linear chain of polymers. The greatest characteristic property of polyrotaxanes is that the macrocycle rings can slide and rotate onto the polymeric chain. This feature gives specific materials, such as the molecular tube formed by cross-linking macrocycles in a single polyrotaxane [28], insulated molecular wires incorporating conductive polymers [29], and a drug-delivery system [30]. Polyrotaxanes, including α-CD and polyethylene glycol (PEG), have been commonly studied due to their facile synthesis and biocompatibility [31,32,33,34].

The main function of polymeric carriers is to pass the drug to the active site, protecting the drug from the interaction with other molecules that cause it to lose its pharmaceutical action. Polymeric carriers avoid the interaction between the drug and proteins which lead to preventing its arrival at the action place. Moreover, polymeric carriers are used as controlled release systems which aim to improve the effectiveness of drug therapy [35]. Sulfaguanidine belongs to the class of sulfonamide drug that is mainly used as an effective antibiotic [36,37]. Since the discovery of sulfonamide-containing antibacterial medicines, the sulfonamide or sulfonyl functional groups have been prominent motifs in medicinal chemistry [38]. The use of sulfonyl or sulfonamide groups in medicinal chemistry is important because it establishes a large class of medicines that are widely used as agricultural and pharmaceutical agents [39,40]. Sulfonamides, as synthetic antifolic agents, have long been used to treat bacterial infections in biological systems, and they have recently attracted a lot of attention in biology and medicine due to their wide variety of biological applications, such as antibacterial [41,42]. Various sulfonyl- or sulfonamide-bearing heterocycles, such as quinazolinones, oxazoles, benzimidazole, thiazole, and pyridazine, have been successfully evolved and used in facilities with sulfadiazine, sulfachlorpyridazine, sulfathiazole, and sulfisoxazole, displaying excellent antibacterial activities [43]. Due to the inadequacy and even loss of resistance of existing antimicrobials against new and emerging bacterial pathogens, novel, less-toxic, and profoundly effective antimicrobial agents were expected to be urgently developed [44]. Moreover, it is also reported that sulfaguanidine prevents infections that cannot be determined by natural and clinical studies [45]. Sulfaguanidine was poorly absorbed by adult rats but was efficiently absorbed by immature gastrointestines of neonatal rats [46]. Poorly absorbed sulfonamides are used in the treatment of tropical sprue [47]. To increase stability, solubility, and to prevent aggregation, functionalizing agents such as polyethylene glycol (PEG) were generally used to increase binding with silver nanoparticles [48]. In literature, most of the searches were concerned with the study of sulfaguanidine at the quantum mechanical level, and the molecule was studied experimentally and theoretically [49]. In addition, a few studies were interested in the increase of the solubility and the bioefficiency of sulfaguanidine [50,51]. Sulfaguanidine was detected by measurements of electrochemical impedance spectroscopy (EIS) and differential pulse voltammetry (DPV). Currently, a simple and highly sensitive approach is performed to synthesize a selective sulfaguanidine sensors based on a molecularly imprinted electropolymer of acrylamide [52].

Incorporation of inorganic nanoparticles (NPs) such as Ag, Pd, CaO, Co_3_O_4_, TiO_2_, ZnO_2_, CuO, MgO, and others into the polymers, which are called nanocomposite polymers, not only dramatically changes their physical properties but also applies new functionalities [53,54,55,56,57,58,59,60,61,62]. Polymeric nanocomposites can be represented as core–shell-type NPs wherein the inorganic core may be either metal and/or a metal–oxide NP, and the organic shell can be a polymer. Thus, one can design a new functional material that may have promising properties including elastic modulus, optical transparency, magnetic, strength, electronic, and even biological performance. However, in fact, many metal–oxide NPs are expensive, and some are also toxic, hindering the industrial revolution [63]. While TiO_2_, in particular, is cost-effective and nontoxic [64], this has been approved by the US Food and Drug Administration (FDA) for use in food and medicine [65]. Moreover, TiO_2_–nanostructures have high surface area and great stability, making them very suitable for many biomedical applications and drug formulation [66]. In addition, recent studies have proven that TiO_2_–NPs and their derivatives are safe and inert materials when exposed to the human body [67].

**Scheme 1:**
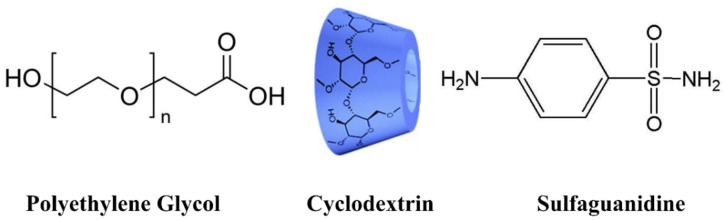
Chemical structure of the compounds used.

In focus of this, the current study aims to design a novel type of polymeric structure that may help improve the potency of a drug. Therefore, three new PEG-based polymers (PEG/α-CD) (**1**), titania nanocomposites (PEG/TiO_2_NPs) (**2**), and pseudopolyrotaxane-titania-nanocomposite (PEG/α-CD/TiO_2_NPs) (**3**) have been synthesized as novel alternative antibiotic strategies against antibiotic-resistant microorganisms. Subsequently, they were used as drug carriers for sulfaguanidine as PEG/α-CD/Drug (**4**), PEG/TiO_2_NPs/Drug (**5**), and PEG/α-CD/TiO_2_NPs/Drug (**6**). The results of this work indicated that this combination significantly modulated the physicochemical properties of sulfaguanidine, including pharmacokinetic response, solubility, and tissue penetration. Thus, it becomes clear that the inclusion of nanopolymers in cyclodextrins has unique pharmaceutical, biomedical, and environmental applications. The chemical compositions of the compounds that were used in this work are illustrated in Scheme 1.

## 2. Experimental

### 2.1. Chemicals and Reagents

α-Cyclodextrin (α-CD), polyethylene glycol (400), and sulfaguanidine were purchased from Alfa Aesar GmbH & Co KG. Dimethylformamide (DMF) was supplied by Aldrich, Milwaukee, WI, USA. All chemicals were used as received without additional purification.

### 2.2. Preparation of TiO_2_ Nanorods

TiO_2_ nanorods were prepared hydrothermally at temperature of 140 °C. The synthesized procedure followed four steps. Briefly, 2 g of commercial TiO_2_ was mixed with 80 mL of 10 M NaOH under stirring for 10 min. The mixture was then transferred to a Teflon-lined autoclave and sintered at 140 °C for 24 h, and finally cooled down to room temperature. The obtained products were collected and washed with diluted HCl (pH 1.6). The product was washed several times with distilled water until the pH 7. As a final step, the product was annealed at 400 °C for 2 h.

### 2.3. General Procedure for the Synthesis of PEG-Based Polymers

#### 2.3.1. Synthesis of Pseudopolyrotaxane PEG/α-CD (**1**)

Polyethylene glycol (1.5 mL, 3.75 mmole) was dissolved in 10 mL DMF, α-Cyclodextrin (5 g, 5.14 mmole) was also dissolved in 15 mL DMF, and then they were mixed. The reaction mixture was stirred for 20 h at 70 °C. The precipitate formed was filtered off, washed with distilled water, and dried to produce the inclusion polymer complex (**1**) as white precipitate (84% yield, m.p. over 300 °C).

#### 2.3.2. Synthesis of PEG/TiO_2_ NPs Composite (**2**)

A mixture of PEG polymer (1.5 g) and of TiO_2_ NPs (0.1 g) in DMF (30 mL) was stirred at room temperature for approximately 12 h, then the formed hydrogel was poured in a Petri dish and left to dry at room temperature.

#### 2.3.3. Synthesis of Pseudopolyrotaxane Composite PEG/α-CD/TiO_2_ (**3**)

First, 1.5 mL of 3.75 mmol Polyethylene glycol (PEG) was dissolved in 10 mL DMF, 5 g of 5.14 mmol α-Cyclodextrin was also dissolved in 20 mL DMF, and then they were mixed. The reaction mixture was stirred for 20 h at 70 °C until a white precipitate was formed, indicating the formation of inclusion complex, then 0.1 g TiO_2_ NPs was added to the reaction mixture and followed by stirring for 12 h. Finally, the formed hydrogel was poured in a Petri dish and allowed to dry at room temperature as a white precipitate (92% yield, and m.p. over 300 °C).

#### 2.3.4. Preparation of PEG/α-CD/Drug (**4**)

Sulfaguanidine (drug) was loaded onto the pseudopolyrotaxane PEG/α-CD by using the colloidal tectonic approach in which 0.4 g of the inclusion complex was dissolved in 30 mL distilled water to form stable hydrogel. Then, 0.2 g of the drug was dissolved in 10 mL distilled water then added into the reaction mixture at room temperature. The reaction mixture was stirred for 10 h until a homogenous solution was obtained. The formed emulsion was poured into a Petri dish and left to dry at room temperature to obtain a white solid.

#### 2.3.5. Preparation of PEG/TiO_2_/Drug (**5**)

Sulfaguanidine was loaded onto the polymer composite PEG/TiO_2_ by two steps: firstly, PEG (1 g) was dissolved in 30 mL distilled water, then TiO_2_ (0.1 g) was dissolved in DMF (5 mL), then added to PEG solution with stirring for 24 h, until the formation of composite. Sulfaguanidine (0.2 g) was dissolved in 10 mL distilled water, then added to the reaction mixture at 60 °C with stirring for 12 h. After that, the obtained emulsion was poured in a Petri dish and left to dry at room temperature to obtain white solid.

#### 2.3.6. Preparation of PEG/α-CD/TiO_2_/Drug (**6**)

Pseudopolyrotaxane composite (0.4 g) was dissolved in 40 mL distilled water to form good hydrogel. Sulfaguanidine (0.2 g) was dissolved in 10 mL distilled water, then added to the reaction mixture with continuous stirring for 15 h at 60 °C. The formed hydrogel was poured into a Petri dish and left to dry at room temperature to obtain a white precipitate.

### 2.4. Characterization

The chemical structures of the fabricated polymers were examined by FT-IR spectroscopy (Shimadzu-IR 408 spectrometer, Tokyo, Japan) in the range of 4000–400 cm^−1^. The IR spectra were obtained against a KBr disc background at room temperature. Chemical composition of the obtained pseudopolyrotaxane was established with proton nuclear magnetic resonance (^1^H-NMR). The ^1^H-NMR spectra were recorded at 25 °C using a Bruker AM-400 NMR spectrometer at 400 MHz. α-CD and pseudopolyrotaxane were respectively dissolved in DMSO-d_6_ solution (Sigma-Aldrich, Burlington, MA, USA). A scanning electron microscope (SEM) (JEOL SEM model JSM-5500-Japan) was used to observe the morphological structures of the obtained materials with accelerated voltage 10 kV. A computerized Analytik Jena SPECORD 200 Plus spectrophotometer as used for studying the optical properties at room temperature in the wavelength range 190–1100 nm. The reproducibility of the data was checked by measuring several specimens.

### 2.5. Antibacterial Tests

Antibacterial tests were performed at the Botany and Microbiology Department, Faculty of Science, South Valley University, Qena, Egypt.

In vitro antibacterial activity tests of all the involved target compounds, including PEG/α-CD (**1**), PEG/TiO_2_ (**2**), PEG/α-CD/TiO_2_ (**3**), PEG/α-CD/Drug (**4**), PEG/TiO_2_/Drug (**5**), PEG/α-CD/TiO_2_/Drug (**6**), and pure sulfaguanidine, against *E. coli*, *Pseudomonas aeruginosa,* and *Staphylococcus aureus* (MRSA) strains were performed against *E. coli* and *Pseudomonas aeruginosa*, both Gram-negative bacteria, and *Staphylococcus aureus* (MRSA), a Gram-positive bacteria. A total of 0.6 g of each compound was dissolved in 1 mL of dimethyl sulfoxide (DMSO) and completed with distilled water to 10 mL to avoid the inhibitory effect of DMSO (10-fold dilution) [68]. Overnight culture of *Escherichia coli, Pseudomonas aeruginosa,* and *Staphylococcus aureus* was adjusted to 0.001 OD_595_ in tryptic soy broth (TSB). A total of 100 µL bacterial growth was placed into 96-well plates with different amounts of each compound (5–50 µL). After 24 h incubation at 37 °C, the MIC was the lowest concentration inhibiting bacterial growth. To confirm bacterial growth inhibition and lack of metabolic activity, 40 µL of *p*-iodonitrotetrazolium violet (INT) (0.2 mg mL^−1^, Sigma-Aldrich) was added to the microplate wells and reincubated at 37 °C for 30 min [69]. The MIC in the INT assay was defined as the lowest concentration that prevented color change, as described earlier [70]. The minimum bactericidal concentration (MBC) test was also performed. The bactericidal effect was defined as a 99.9% decrease in CFU (three logs) in the starting inoculum during 24 h of incubation. The MBC was determined by transferring 50 µL from each well of overnight MIC plates to sterile tryptic soy agar (TSA) fresh plates. Viable colonies were counted after 24 h at 37 °C. The limit of detection (LOD) for this assay was 10 cfu mL^−1^ [71].

### 2.6. Static Biofilm Assay

Static biofilm was performed in microtiter plates by crystal violet staining, as described elsewhere [72]. The respective strains were grown overnight on tryptic agar plates (Oxoid^®^). The growth was suspended in tryptic broth, adjusted to an OD_595_ of 0.02, then 130 µL of each strain dilution was placed in a 96-well microtiter plate (U Bottom, Sterilin) for 24 h at 37 °C, followed by addition of 30 μL of each polymer to assess their antibiotic efficacy. Wells were subsequently washed with distilled water and biofilm was stained with 0.1% crystal violet for 10 min. Then, the wells were washed again with distilled water and the contents dissolved in ethanol (96%). The OD_595_ was measured with an Infinite^®^ F50 Robotic (Ostrich) Microplate Reader for biofilm quantification.

## 3. Results and Discussion

### 3.1. Chemistry

**Scheme 2:**
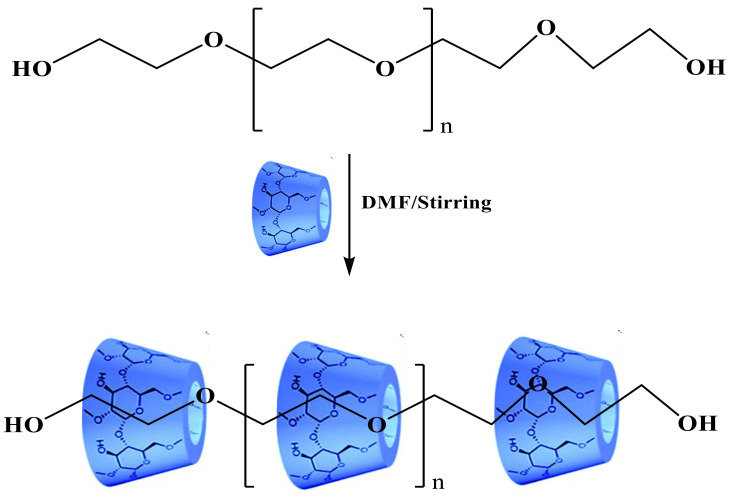
Synthetic route for the synthesis of the inclusion complex PEG/α-CD (**1**).

**Scheme 3:**
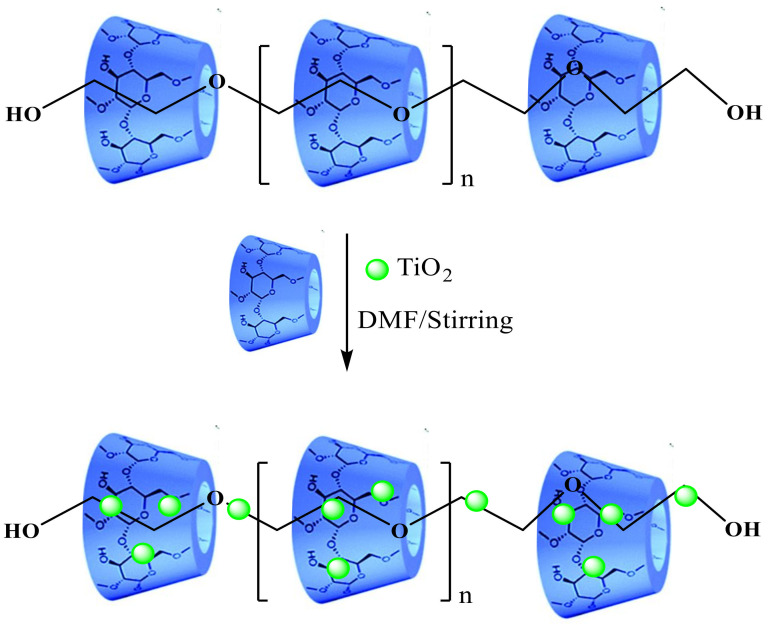
Synthetic route for the synthesis of the inclusion complex PEG/α-CD/TiO_2_ (**3**).

**Scheme 4:**
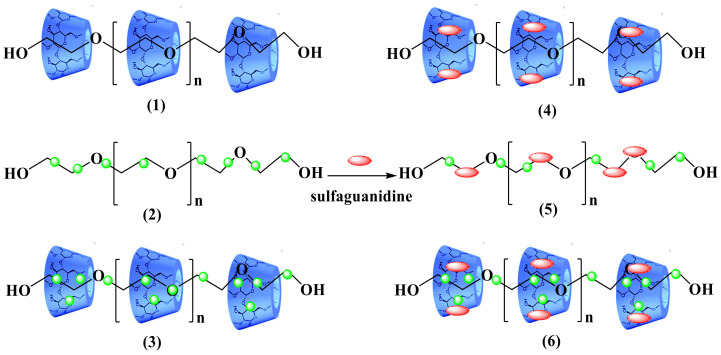
Synthetic route for the loading sulfaguanidine onto the polymers (**1**–**3**).

Scheme 2 illustrates the mechanism that has been proposed for the synthesis of the inclusion complex PEG/α-CD (**1**). It can be seen that the α-CD macrocycle is inserted onto the PEG chain by threading method via formation of hydrogen bonds and hydrophobic interaction. This inclusion complex is more stable due to PEG having more hydrophobic chains in comparison with the solvent used (DMF). Thus, selective threading may occur when the polymer structure consists of hydrophobic and hydrophilic parts. In addition, α-CD forms a crystalline inclusion complex with PEG. The formation of the inclusion complex depends strongly on structure, molecular weight, and geometry of the PEG. Pseudopolyrotaxane composite PEG/α-CD/TiO_2_ (**3**) was designed by threading method in the presence of TiO_2_ NPs (Scheme 3). Loading the sulfaguanidine onto the polymers (**1**–**3**) is shown in Scheme 4, in which sulfaguanidine and the obtained polymers are dispersed in water. The reaction mixture is heated to obtain viscous and translucent liquid, and the solution is left to give solid precipitate, separated, and dried to give (**4**–**6**), respectively.

### 3.2. FT-IR-Studies

**Figure 1:**
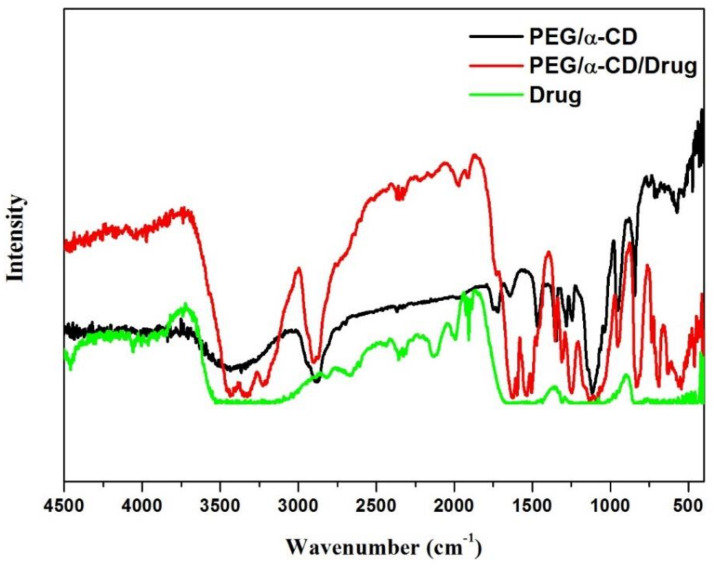
FT-IR spectra of sulfaguanidine drug (green), PEG/α-CD (black), and PEG/α-CD/Drug (red).

Pseudopolyrotaxane (**1**) was synthesized as represented in Scheme 1, and the vibration characteristics of chemical functional groups were studied by FT-IR spectroscopy. FT-IR spectra of (**1**) shows several characteristic bands at 3401 cm^−1^ for hydroxyl groups, with a characteristic band due to CH aliphatic at 2937 cm^−1^. The absorption band of the hydroxyl groups is lighter compared to those of the pure α-CD (Figure 1). In addition, the ν[OH] symmetric stretching was shifted to lower frequency and ν[CH-aliphatic] was shifted to higher frequency compared to those in pure α-CD. In addition, the absorption band for ν[C–O-C] was shifted to higher frequencies. Table 1 indicates the differences in the absorbance bands of pure α-CD and pseudopolyrotaxane (**1**). The shifting that occurs in absorbance bands could strongly illustrate the formation of an inclusion complex between PEG and the macrocyclic molecules of α-CDs. The enhancement in frequencies is due to the insertion of the PEG chain through the electron-rich cavity of the cyclodextrin rings [73,74,75,76]. In contrast, the decreasing in frequencies is due to the creation of van der Waals forces, hydrogen bonds between the adjacent α-CDs and the hydrogen bonds between the hydroxyl groups of α-CDs and the oxygen ether linkages in PEG. These results can be taken as evidence for the formation of the inclusion complex between α-CD and PEG as a result of the change in the frequency of the α-CD functional groups after formation of the inclusion polymer complex.

The FT-IR spectrum of sulfaguanidine showed a band at 3144 cm^−1^ correlating to the amino groups (NH_2_) and a band at 1567 cm^−1^ correlating to the imine (C=N) group. The absorption band at 1367 cm^−1^ characterized the stretching vibration of the SO_2_ group (see Figure 1). The differences between absorbance bands of pure drug and pseudopolyrotaxane/drug (**4**) are summarized in Table 2. There is a difference in the intensity of sulfaguanidine before and after being loaded onto pseudopolyrotaxane (**1**). This shift is due to formation of hydrogen bonds between amino groups in sulfaguanidine and the hydroxyl groups α-CDs in addition to the hydrophobic interaction and van der Waals forces. These actions could strongly confirm the loading drug onto the pseudopolyrotaxane (**1**). The increase in frequencies is due to the insertion of the aromatic ring through the cavity of α-CDs. On the other hand, the decreasing in frequencies is due to the formation of van der Waals forces and hydrogen bonds between the hydroxyl groups of α-CDs and amino, the sulphonyl group of the drug.

**Figure 2:**
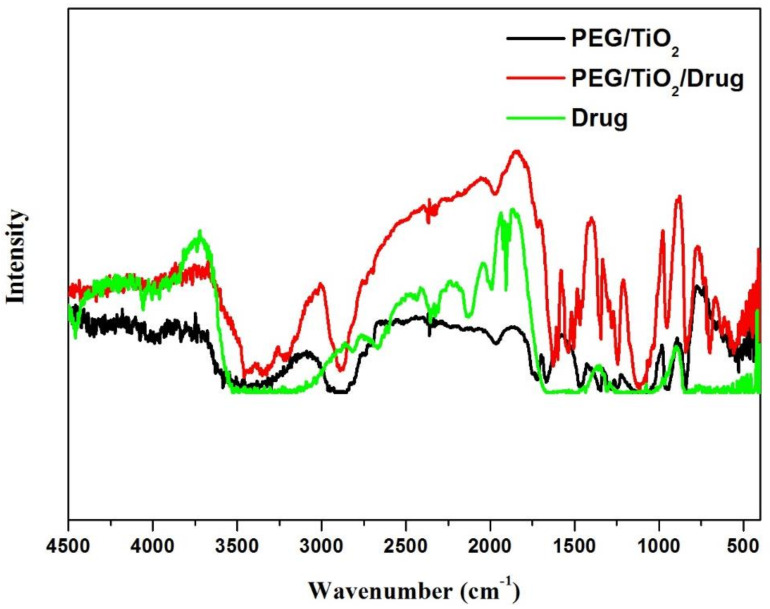
FT-IR spectra of the drug (green), PEG/TiO_2_ (black), and PEG/TiO_2_/Drug (red).

The FT-IR spectra of sulfaguanidine (drug), PEG polymer doped with TiO_2_ NPs (**2**), and PEG composite/drug (**5**) are characterized in Figure 2. The FTIR spectrum of PEG/TiO_2_ (**2**) shows a broad absorption band at 3450 cm^−1^ corresponding to the stretching vibrations of hydroxyl (OH) groups, that is shifted and decreases in intensity 3326 after loading the drug. The absorption band at 2852 cm^−1^ is attributed to symmetric stretching of aliphatic C–H groups of PEG, which is shifted and increases in intensity 2900 cm^−1^ upon loading of the drug. The intense peak at 690.39 cm^−1^ is indicative of the Ti–O stretching band that is characteristic for TiO_2_ [77]. In addition to the FT-IR spectrum, the pure drug shows increasing and decreasing in the intensity of the absorption bands. The absorption band of amino groups was shifted towards lower wave numbers and decreased in their intensities as a result of the formation of hydrogen bonds between the oxygen atoms of the ether linkage of PEG. The differences between absorption bands of drug before and after loading are summarized in Table 3. The shift that occurred in the intensity indicates the strong interaction between the functional groups in the drug and the polymer composite (**2**).

**Figure 3:**
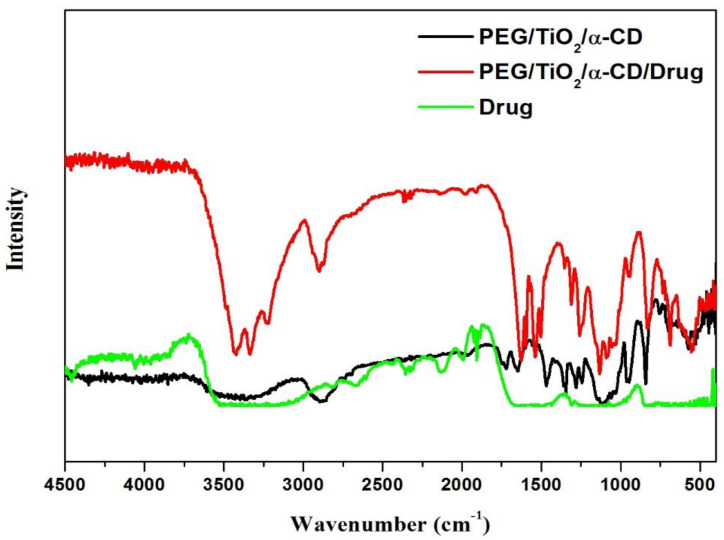
FT-IR spectra of the drug (green), PEG/TiO_2_/α-CD (black), and PEG/TiO_2_/α-CD/Drug (red).

Figure 3 shows the FTIR spectra of pseudopolyrotaxane composite (**3**), sulfaguanidine (drug), and pseudopolyrotaxane composite/drug. The FTIR spectrum of PEG/TiO_2_/α-CD (**3**) displays a broad absorption band at 3417 cm^−1^ corresponding to the stretching vibrations of hydroxyl (OH) groups. The absorption band at 2898 cm^−1^ is due to symmetric stretching of aliphatic C–H groups of PEG. The absorption band at 691.35 cm^−1^ corresponds to TiO_2_. The FT-IR spectrum of the pure drug illustrates increasing and reducing in the intensity of their absorption bands after their loading on the pseudopolyrotaxane composite (**3**). The differences in the absorption bands are summarized in Table 4. The change in the intensity indicates good evidence for the loading of the drug onto the polymer (**3**). Table 5 shows the change in the absorption bands of pseudopolyrotaxane (**1**) after formation of the pseudopolyrotaxane composite (**3**). Thus, FTIR spectra of (**3**) demonstrates a band at 3417 cm^−1^ for hydroxyl groups, a characteristic band due to CH aliphatic at 2898 cm^−1^, and the appearance of a characteristic stretching band for TiO_2_ at 691.35. The ν[OH] symmetric stretching was shifted to higher frequency, and ν[CH-aliphatic] was shifted to lower frequency. In addition, the absorption band for ν[C–O-C] was shifted to higher frequencies. This behavior is due to the insertion of TiO_2_ through the matrix and formation of pseudopolyrotaxane composite.

### 3.3. ^1^H-NMR Analysis

**Figure 4:**
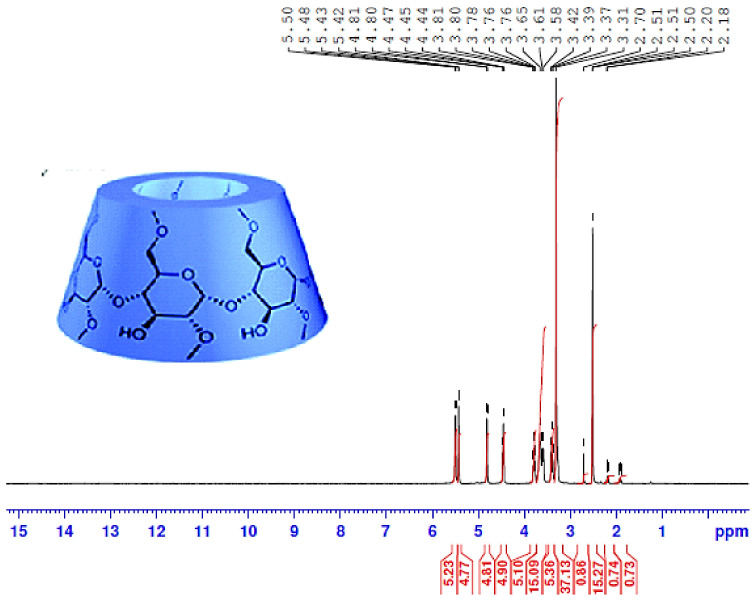
^1^H-NMR spectra for α-CD in DMSO-d_6_.

**Figure 5:**
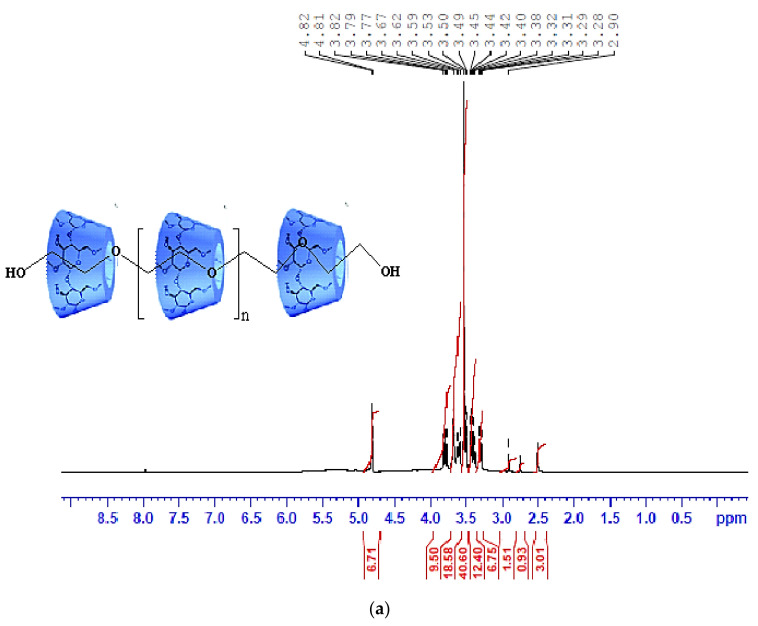

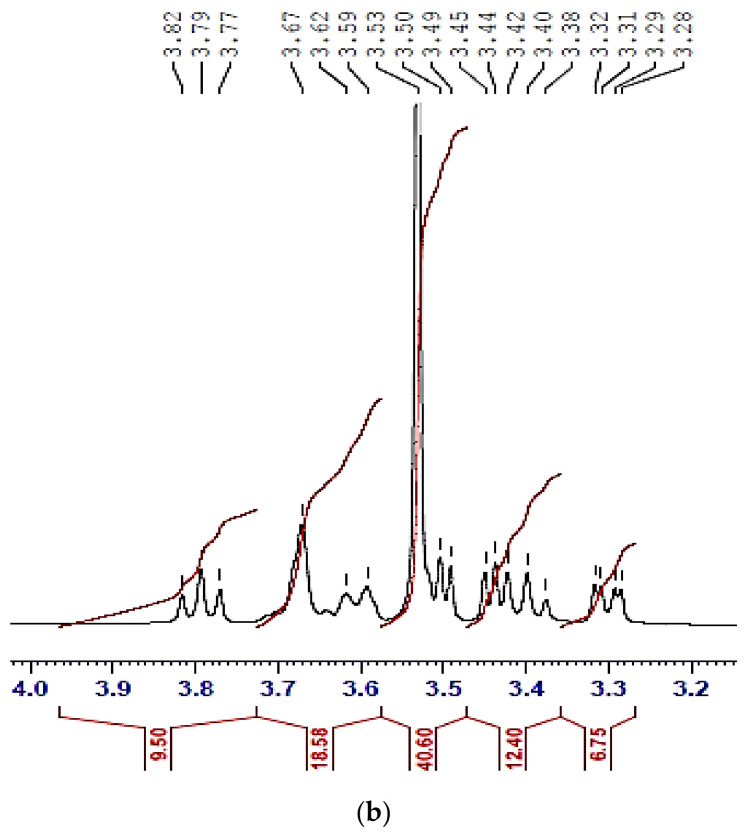
(**a**) ^1^H-NMR spectra for PEG/α-CD in DMSO-d6, and (**b**) expanded section of aliphatic protons in α-CD.

^1^H-NMR spectra of pure α-CD and the inclusion complex PEG/*α*-CD (**1**) are displayed in Figure 4 and Figure 5. The peak at 3.5 ppm was the aliphatic methylene protons of the PEG. The proton signals of *α*-CDs were localized from 3.28 to 4.82 ppm. Comparison of the integral for the α-CD (1H) peak and that of the methylene group on PEG introduces two monomer units inserted into the α-CD ring. The stoichiometric ratios are always 2:1 (monomer unit: α-CD). Table 6 shows the chemical shifts observed for H-1, H-2, H-3, H-4, H-5, and H-6 for pure α-CD and its inclusion polymer complex PEG/*α*-CD (**1**). It is observed that the values of chemical shift for protons of α-cyclodextrin after formation of inclusion complex decreased and moved more up field due to the formation of hydrophobic–hydrophobic interaction between the aliphatic chain of PEG and the hydrophobic sites in the cavity of α-CD, indicating the formation of an inclusion polymer complex.

### 3.4. Surface Morphology

**Figure 6:**
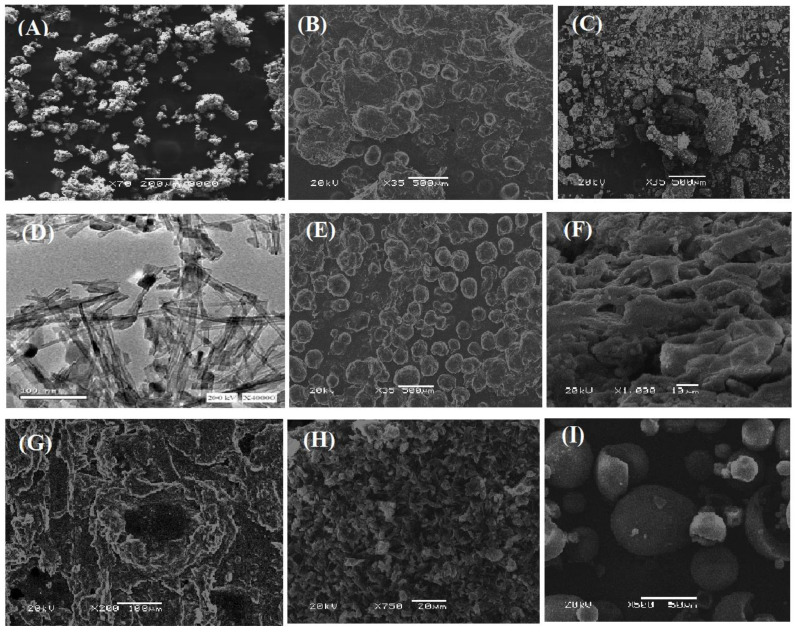
SEM images of the sulfa drug and prepared antimicrobial agents based on PEG. (**A**) α-CD, (**B**) PEG/α-CD, (**C**) PEG/α-CD/Drug, (**D**) TiO_2_ NPs, (**E**) PEG/TiO_2_, (**F**) PEG/TiO_2_/Drug, (**G**) PEG/α-CD/TiO_2_, (**H**) PEG/α-CD/TiO_2_/Drug, (**I**) Sulfaguanidine.

The morphological structure of α-CD (A), PEG/α-CD (B), PEG/α-CD/Drug (C), TiO_2_ NPs (D), PEG/TiO_2_(E), PEG/TiO_2_/Drug (F), PEG/α-CD/TiO_2_ (G) PEG/α-CD/TiO_2_/Drug (H), and sulfaguanidine (I) are elucidated in Figure 6. These images show a complete difference between them, and it was found that the surface appearances were changed upon reaction, as compared to the fibrous nature of sulfaguanidine surface. The surface morphology structure of PEG/α-CD/Drug (C) is different from α-CD (A), PEG/α-CD (B), and sulfaguanidine (I); this gives good indication for the formation of an inclusion complex. In addition to successfully loading the drug onto the pseudopolyrotaxane, PEG/α-CD/Drug (C) appeared as amorphous slides. Furthermore, the SEM image PEG/TiO_2_/Drug (F) is different than TiO_2_ NPs (D) and PEG/TiO_2_ (E). TiO_2_ NPs (D) presented as flat, PEG/TiO_2_ (E) was localized as the coherent structure, whereas PEG/TiO_2_/Drug (F) matrix was found to be a regular, circular, and poreless surface. Thus, this may be due to the inserting of TiO_2_ NPs into the interplane spacing of the matrix. In addition, in SEM images of PEG/α-CD/TiO_2_/Drug (H), their morphology structure is completely different from PEG/α-CD/TiO_2_ (G) and drug (I), as well having regular structure similar to peels.

### 3.5. Optical Properties

**Figure 7:**
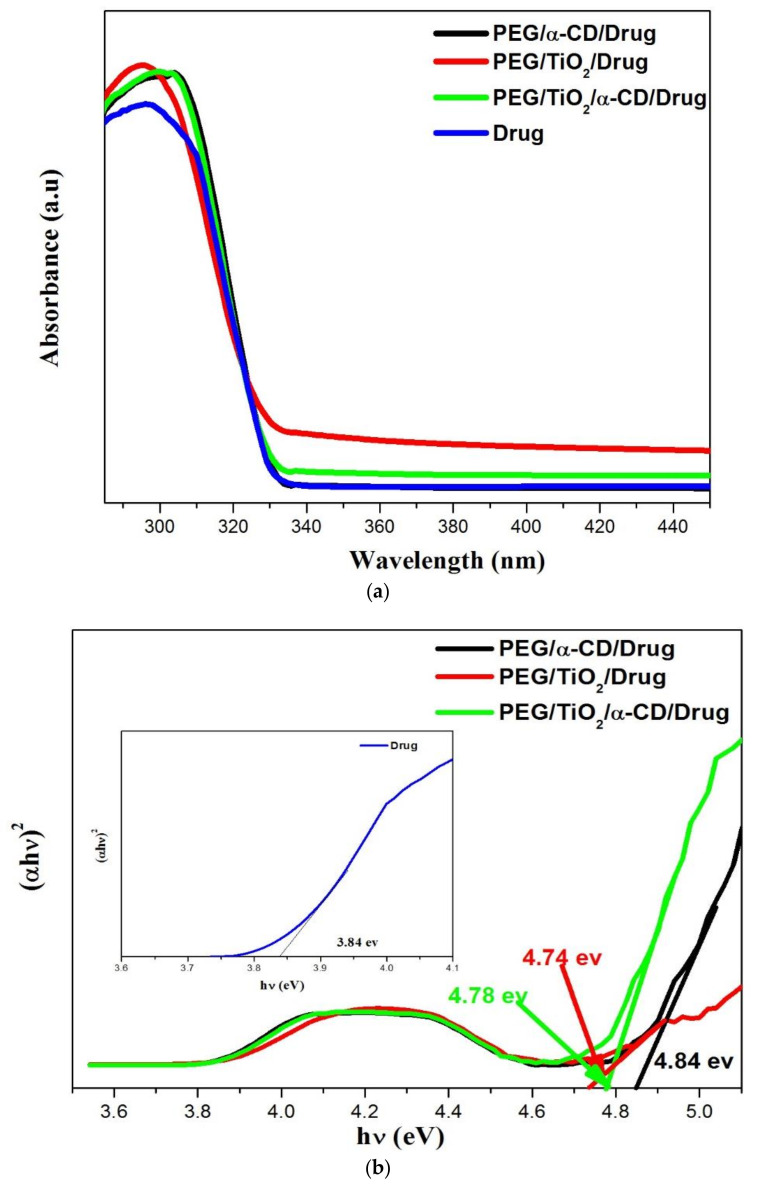
(**a**) UV–Vis absorption spectra of the drug before and after loading on the polymers, and (**b**) the corresponding estimated values for the energy gap.

The optical properties of the drug were recorded before and after loading in the three nanopolymers (**1**–**3**) using UV spectroscopy at 25 °C. Figure 7 shows a comparison of the absorption spectra of pure drug, PEG/α-CD/Drug, PEG/TiO_2_/Drug, and PEG/α-CD/TiO_2_/Drug. The UV–Visible spectrum of the drug displays a main characteristic peak at 296 nm. These peaks are assigned to the n–л* transition of imine (–N=C) bond of the drug. Upon loading of the drug into pseudopolyrotaxane (**1**), a slight blue shift was observed in the characteristic peak from 296 to 304 nm. Similarly, a blue shift also occurred from 296 to 298 nm and from 296 to 306 nm by loading the drug into the polymer composite (**2**) and pseudopolyrotaxane composite (**3**), respectively. The band gap energy (E_g_) of the drug was estimated before and after loading in polymers (**1**–**3**) according to Tauc’s formula. As depicted in Figure 7b, values of 3.84, 4.84, 4.74, and 4.78 eV were obtained for drug, PEG/α-CD/Drug, PEG/TiO_2_/Drug, and PEG/α-CD/TiO_2_/Drug, respectively.

### 3.6. Antimicrobial Performance Evaluation

A comparison of the antimicrobial effect of sulfaguanidine before and after loading of prepared polymers versus some Gram-negative and Gram-positive bacteria was studied and summarized in Table 7. Inspection of the data displayed in Table 7, it can be seen that pseudopolyrotaxane PEG/αCD (**1**) has exhibited an excellent effect against both Gram positive and Gram-negative bacteria with MIC and MBC ranging from 8.5–17 mg mL^−1^, and the mixture inhibiting biofilm had a percentage of 41.2–52.5%. Conversely, the antibacterial efficacy and antibiofilm behavior completely diminished when PEG/α-CD was used as the carrier of a sulfaguanidine drug, PEG/α-CD/Drug (**4**). The polymer composite PEG/TiO_2_ (**2**) demonstrated good efficacy on all tested bacteria, with MIC and MBC ranging from 10–20 mg mL^−1^ and an antibiofilm inhibition percent of 34.8–39.5%. It is worth noting that when PEG/TiO_2_ combined with sulfaguanidine PEG/TiO_2_/Drug (**5**), antibacterial efficacy of the mixture was increased, with MIC and MBC ranging from 7–14 mg mL^−1^ and the antibiofilm inhibition percentage ranging between 44.5–53.9%. The effect of pseudopolyrotaxane composite PEG/αCD/TiO_2_ (**3**) was effective only against Gram-negative bacteria, with MIC and MBC at 6 mg mL^−1^ and the inhibition percentage against Gram-negative bacteria only ranged from 60.7–73.2%. On the other hand, when the pseudopolyrotaxane composite was used as carrier for sulfaguanidine PEG/αCD/TiO_2_/Drug (**6**), the efficacy decreased, and the value of MIC and MBC was 20 mg mL^−1^, and inhibition percentage ranged from 33.3–37.3%. Interestingly, no effect against both Gram-negative and Gram-positive bacteria was recorded for sulfaguanidine alone (SULFA).

PEGs are listed by the FDA as generally safe compounds, due to their low toxicity, low immunogenicity and antigenicity, low flammability, biodegradability, and rapid excretion after administration to living organisms [78]. Therefore, they are used in various fields, especially in the medical and pharmaceutical sectors, where conjugates of PEGs and proteins/drugs have been developed in order to extend circulation times in the blood [79]. In the current study, PEG/α-CD (**1**) exhibited a good efficacy as antibacterial and antibiofilm against all tested bacterial strains (Table 7). CDs offer several innovative strategies against microbial infection including not only reducing the resistance of the known antibiotics via complexation, but also decreasing the cell-to-cell communication (quorum sensing), inhibiting bacterial and viral infections via cholesterol depletion, and plugging the pores of the pathogens by CDs [80,81,82,83]. Antibiotic resistance is on the rise, and high repetition rates are expected to exacerbate the problems that these common infections pose to society [55,84]. Therefore, the treatment of bacterial infections has become a major source of concern due to the emergence of bacterial resistance to traditional antibiotics [85]. As a result, the production of novel and efficient bactericidal agents is crucial in clinical practice. Intriguingly, metallic nanoparticles (NPs) have been shown to be a promising antibiotic substitute. NPs interact with cellular organelles and biomolecules such as DNA, enzymes, ribosomes, and lysosomes, influencing cell membrane permeability, protein activation, gene expression, and oxidative stress [48]. In the current study, PEG/TiO_2_ (**2**) was effective against all tested bacterial strains, in close agreement with other previous works [86,87]. As PEG/TiO_2_ NPS are smaller in size, they are expected to have a better chance of interacting with bacteria or better access to endogenous bacterial proteins. The question is, what is the effect of adding a sulfa drug to the composition of compound (**2**)? The results indicated that this combination significantly modifies the properties of the sulfaguanidine and increases antibacterial and antibiotic efficacy when mixed with PEG/TiO_2_/Drug (**5**), compared to using the pure drug (SULFA) or even using a mixture of PEG/TiO_2_ (**2**). Moreover, the efficacy was much higher in the use of PEG/α-CD/TiO_2_ (**3**) polymer, but the effect only occurred on Gram-negative strains; even after the addition of a sulfaguanidine to the complex, i.e., PEG/α-CD/TiO_2_NPs/Drug (**6**), the efficacy was also only good against Gram-negative strains. Although sulfaguanidine is poorly absorbed from the gut, it is well suited for the treatment of bacillary dysentery and other enteric infections [88]. Thus, the significant improvement in the efficacy of sulfaguanidine when mixed with the currently used polymer may be due to the enhanced drug absorption properties. In fact, bacterial biofilms have become a major threat to human health. They have a high level of antibiotic resistance and are hosts to immune response stimulants [88,89], as well as being essential in the pathogenesis of several chronic human infections [90]. A biofilm is a complex community of bacteria attached to a surface or interface enclosed in an exopolysaccharide matrix, protected from unfavorable antibiotics, host defenses, or oxidative stresses [91]. Interestingly, experimental tests on the new designed compounds showed promising efficacy in biofilm inhibition, and the pseudopolyrotaxane nanocomposite (PEG/αCD/TiO_2_) showed the highest level.

## 4. Conclusions

Three promising novel systems, namely, pseudopolyrotaxane, polymer composite, and pseudopolyrotaxane composite containing PEG were synthesized. The chemical structure and the characterization of the obtained polymers were investigated by FTIR, ^1^H-NMR, SEM, and optical methods. In addition, they were used as drug carriers for sulfaguanidine. Antibacterial and antibiofilm activity of the target compounds confirmed the ability of these compounds as a new preventive measure against multidrug-resistant strains, also showing enhancement of the efficacy of the drug. The results confirmed that the inclusion of nanopolymers in cyclodextrin increases the solubility of bad drugs and increases their bioavailability and stability, giving them unique pharmaceutical and biomedical applications.

## Figures and Tables

**Table 1:** FT-IR difference between pure α-CD and PEG/α-CD **(1)**.

Functional Group	Wavenumber, cm^−1^		Δδ
	α-CD	PEG/α-CD (1)	
ν[OH] symmetric	3407.64	3401.81	−5.83
ν[CH-aliphatic]	2927.41	2937.05	+9.64
ν[C–O–C]	1157.08	1159.97	+2.89
ν[OH] bending	1078.01	1080.90	+2.89

**Table 2:** FT-IR difference between pure drug before and after loading upon PEG/α-CD.

Functional Group	Wavenumber, cm^−1^		Δν
	Drug	PEG/α-CD/Drug (4)	
ν[NH_2_] symmetric	3144.37	3129.90	−14.47
ν[CH-aromatic]	3010.33	3016.06	+5.73
ν[SO_2_]	1367.28	1374.99	+7.71
ν[C=N]	1567.84	1550.37	−17.47

**Table 3:** FT-IR difference between pure drug before and after loading upon PEG/TiO_2_.

Functional Group	Wavenumber, cm^−1^		Δν
	Drug	PEG/TiO_2_/Drug (3)	
ν[NH_2_] symmetric	3144.37	3130.86	−13.51
ν[CH-aromatic]	3010.33	3019.97	+9.64
ν[SO_2_]	1367.28	1397.17	+29.89
ν[C=N]	1567.84	1579.41	+11.57

**Table 4:** FT-IR difference between drug before and after loading on pseudopolyrotaxane composite.

Functional Group	Wavenumber, cm^−1^		Δν
	Drug	PEG/α-CD/TiO_2_/Drug (6)	
ν[NH_2_] symmetric	3144.37	3136.56	−7.81
ν[CH-aromatic]	3010.33	3005.51	−4.62
ν[SO_2_]	1367.28	1382.71	+15.43
ν[C=N]	1567.84	1577.48	+9.64

**Table 5:** FT-IR difference between pseudopolyrotaxane (**1**) and pseudopolyrotaxane composite (**3**).

Functional Group	Wavenumber, cm^−1^		Δδ
	PEG/α-CD (1)	PEG/TiO_2_/α-CD (3)	
ν[OH] symmetric	3401.81	3417.24	+15.43
ν[CH-aliphatic]	2937.05	2898.15	−38.9
ν[C–O–C]	1159.97	1161.90	+1.93
ν[OH] bending	1080.90	1079.94	−0.96

**Table 6:** Chemical shifts (ppm) for the protons of pure *α*-cyclodextrin and inclusion polymer complex PEG/α-CD (1).

Compound	H-1	H-2	H-3	H-4	H-5	H-6
α-CD	4.81	3.65	3.78	3.61	3.76	3.81
PEG/α-CD (1)	4.82	3.44	3.62	3.28	3.49	3.79
Δδ	0.01	0.21	0.16	0.33	0.27	0.02

**Table 7:** Comparison of the antimicrobial effect of sulfaguanidine before and after loading to prepared polymers versus some Gram-negative and Gram-positive bacteria.

Strains	*E. coli*			*Ps. aeruginosa*			*S. aureus*		
Compound	MIC	MBC	BIP	MIC	MBC	BIP	MIC	MBC	BIP
PEG/αCD (**1**)	17 ± 1	17 ± 1	52.5	17±	17±	41.2	8.5 ± 0.5	8.5 ± 0.5	15.5
PEG/αCD/Drug (**4**)	-ve	-ve	-ve	-ve	-ve	-ve	-ve	-ve	-ve
PEG/TiO_2_ (**2**)	20 ± 2	20 ± 2	39.5	20 ± 2	20 ± 2	34.8	10 ± 1	10 ± 1	9.5
PEG/TiO_2_/Drug (**5**)	14 ± 2	14 ± 2	53.9	14 ± 2	14 ± 2	44.5	7 ± 1	7 ± 1	13.8
PEG/αCD/TiO_2_ (**3**)	6 ± 0.5	6 ± 0.5	73.2	6 ± 0.5	6 ± 0.5	60.7	-ve	-ve	-ve
PEG/αCD/TiO_2_/Drug (**6**)	20 + 1	20 + 1	33.3	20 + 1	20 + 1	37.3	-ve	-ve	-ve
Pure Drug (SULFA)	-ve	-ve	-ve	-ve	-ve	-ve	-ve	-ve	−ve

Antibacterial profile of the target compounds against *Escherichia coli (E. coli), Pseudomonas aeruginosa (Ps. aeruginosa)* and *Staphylococcus aureus* (*S. aureus)*. MIC (minimum inhibitory concentration expressed in mg mL^−1^); MBC (Minimum bactericidal concentration expressed in mg mL^−1^); BIP (biofilm inhibiting percentage expressed in %); –ve means the effect was negative, and Drug (SULFA) = sulfaguanidine. MIC and MBC expressed as mean ± sdv.

## Data Availability

Not applicable.
